# A Cross-Sectional Study of Spanish Grindr Users: Sociodemographic Characteristics, Sexual Health Trends, and Attitudes toward HIV Testing

**DOI:** 10.3390/healthcare12171722

**Published:** 2024-08-29

**Authors:** Eduardo Ibáñez-Tomás, Ángel Gasch-Gallén

**Affiliations:** 1 Servicio Aragonés de Salud, Hospital Universitario Miguel Servet, 50009 Zaragoza, Spain; 2 Departamento de Fisiatría y Enfermería, Universidad de Zaragoza, 50009 Zaragoza, Spain; angelgasch@unizar.es; 3 Grupo Enfermero de Investigación en Atención Primaria de Aragón (GENIAPA-GIIS094), Instituto Investigación Sanitaria Aragón—IISA, 50009 Zaragoza, Spain; 4 Grupo Aragonés de Investigación en Atención Primaria (GAIAP-GIIS011), Instituto Investigación Sanitaria Aragón—IISA, 50009 Zaragoza, Spain

**Keywords:** men who have sex with men, sexually transmitted infections, dating app, risk behavior

## Abstract

Men who have sex with men are at an increased risk of acquiring sexually transmitted infections, and although behavioral and contextual interventions have improved, infections continue to spread. Therefore, a new focus on recent trends in sexual health in this population is needed. The aim of this study was to describe the relationship between sociodemographic and behavioral characteristics according to Grindr usage patterns, the prevalence of sexually transmitted infections (STIs), and attitudes toward HIV testing. In January 2020, a cross-sectional study was conducted using a sample of 881 men who have sex with men (MSM) who completed an online questionnaire. We evaluated their Grindr use patterns (moderate or intensive) and explored the associations of these with sociodemographic behavioral characteristics, STIs, and attitudes toward HIV testing. Of 881 participants in total, 587 (66.6%) were intensive Grindr users. Compared to moderate users, these participants reported significantly higher numbers of casual sexual partners (76.2% vs. 23.8%; *p* < 0.001), were more likely to participate in Chemsex (77.3% vs. 22.7%: *p =* 0.031), and had a higher self-reported number of STIs (69.9% vs. 30.1; *p* = 0.046). They also demonstrated better attitudes toward HIV testing (69.7% vs. 30.3%; *p* = 0.045) and perceived themselves to be at moderate risk of HIV (71.2% vs. 28.8%: *p* = 0.048). Moreover, intensive Grindr users were 1.36 times more likely than moderate users to test positive for STIs (95%CI = 1.15–1.91; *p* = 0.048). With some limitations, age, sexual partners, intensive Grindr users, and PrEP uses were associated with the risk of STIs in the sample of Spanish Grindr users studied. As Grindr users are vulnerable to sociodemographic and behavioral factors that determine STIs and HIV infection, mainly among intensive users, it is necessary to highlight the importance of new trends such as online dating apps, PrEP use, substance use in sex, and Chemsex, and these need to be incorporated into online public health strategies.

## 1. Introduction

The development of new Information and Communications Technologies (ICTs) has changed people’s lives, affecting how they relate to others [1,2,3]. The popularization of the internet and excessive smartphone use have brought millions of people closer together and facilitated means of finding partners. Specifically, online dating apps, which are characterized by the use of geolocation-based real-time dating, have grown considerably [4,5,6]. Different studies on dating apps have focused on describing user profiles, usage patterns [7,8,9], the relationship of app use to mental health (e.g., problematic use, use related to dark personality traits) [8,10], and overall relationship health (e.g., sexual risk behaviors, infidelity) [11,12,13,14].

In recent years, dating apps have attracted research interest in the field of public health, resulting in different studies that have focused on describing user profiles and usage patterns [7,8,9]. In this regard, relationships with mental health (e.g., problematic use and abusive personality patterns) [8,10] and changes in relational health (e.g., high-risk sexual behaviors) [11,12,13,14] have been found. Online partner seeking appears to be associated with increased sexual risk behaviors and a higher prevalence of sexually transmitted infections (STIs) [14,15,16,17,18,19]. However, and despite increased interest in factors related to the risk of human immunodeficiency virus (HIV) transmission, data regarding increased HIV acquisition among MSM who use dating apps are lacking [20,21]. Similarly, there is a lack of studies focused on assessing the use of dating apps and their impact on acquiring STIs and user attitudes to HIV testing, as well as on new phenomena such as Chemsex (intentional sexual practices under the effect of psychoactive drugs in a group for a prolonged duration) [22,23] or the use of pre-exposure prophylaxis (PrEP) [24,25] in specific contexts such as Spanish MSM.

Grindr, created in 2009, is the most popular and frequently used dating app aimed mainly at gay, bi, trans, and queer people. It is a mobile geo-social application that enables its users to locate and communicate with other gay, bisexual, and heterosexual men in proximity, as well as transgender individuals and transsexuals. It is estimated to be available in more than 192 countries and has millions of daily users [26].

Given its current relevance, growing importance in human relations, and impact on health, Grindr remains a novel phenomenon requiring further research. To the best of our knowledge, there are no previous studies in the Spanish MSM population. Therefore, the aim of this study was to describe the sociodemographic and behavioral characteristics of Grindr user profiles according to usage patterns and to identify the frequency of STIs and attitudes toward HIV testing among Spanish Grindr users.

## 2. Materials and Methods

### 2.1. Study Design, Participants, and Sample

A cross-sectional descriptive quantitative study based on an online survey in the Spanish MSM population was conducted. The following four inclusion criteria were used: (1) self-identification as a man, (2) an age between 16 and 75 years, (3) being a resident of Spain, and (4) a declaration of sexual relations with other men in the previous 12 months. Exclusion criteria included (1) participants who refused to provide informed online consent and (2) participants who incorrectly filled out the questionnaire or left it incomplete. A convenience sampling method was used.

### 2.2. Instruments and Procedure

An anonymous online survey was developed and used to collect data about sociodemographic characteristics, sexual behavior in the past 12 months, history of STIs, intentions to undergo HIV testing, and perceived risk of HIV. Participants were recruited through Grindr, a mobile geo-social application mainly aimed at a gay audience, which enables its users to locate and communicate with gay and bisexual men and women, as well as transgender individuals and transsexuals, in the vicinity. Through their program “Grindr for equality” (G4E), in January 2020, advertisements were disseminated for this research study for 48 h, encouraging users to participate in the survey. The survey stated that participants could only respond if they were of legal age which, with respect to sexual matters in Spain, is 16 years old. The anonymity and confidentiality of the participants were guaranteed throughout the process.

### 2.3. Measures

#### 2.3.1. Sociodemographic and Behavioral Characteristics

We asked participants about their age, place of birth (Spain or other), education level (college degree and above, vocational training, secondary school, or below), employment (retired, student, employed, or unemployed), monthly income (>1200 EUR, 700–1200 EUR, or ≤700 EUR), cohabitation situation (stable partner, alone, or other, e.g., family-shared apartment) and city size (<10,000; 10,000–100,000; 100,000–500,000; 500,000–1,000,000, or >1,000,000).

Questions also concerned the frequency of Grindr use, which was dichotomized into moderate or intensive use. Intensive users were defined as users who always or almost always used Grindr to find a sexual partner during the past year (>50%), whereas those who never or almost never used Grindr to find a sexual partner (<50%) were considered moderate users.

Sexual roles (active/insertive, passive/receptive, or versatile—insertive and receptive), sexual orientation (heterosexual, bisexual, or gay/homosexual), sexual partners in the past 12 months (regular, casual, or both), Chemsex participation (never or sometimes/usually), sex while under the influence of drugs (no or yes), and PrEP use (never or usually) were also explored.

#### 2.3.2. STIs, Attitudes toward HIV Testing, and Perceived Risk of HIV Infection

The participants were asked if they had ever had STIs (no or yes). Those who answered “yes” were asked which STIs they had presented. We investigated their attitudes to HIV testing by the question “Have you been tested for HIV?” (yes, in the last 12 months, yes but not in the last 12 months, or never). Those who answered “yes” were directed to a multiple-choice segment of the questionnaire about the reasons why they underwent HIV testing (e.g., “I had a risky practice and wanted to have it done”). Participants who answered, “never have been tested for HIV” were referred to a multiple-choice segment of the questionnaire to explain why (e.g., “I have not had any risky practice so far”). They were also asked about their perceived risk of HIV infection (“At low risk of HIV infection” or “At moderate risk of HIV infection”) and what the reasons for this perception were (e.g., “I have not had unprotected sex” or “I have been unable to make an appointment for HIV testing”).

### 2.4. Statistical Analyses

In the first phase of the analysis, descriptive statistics were conducted to describe the demographic and behavioral characteristics of the participants, as well as STIs, attitudes toward HIV testing, and perceived risk of HIV infection. Tables of frequency and proportions were drawn for qualitative variables, and measures of centralization were established for quantitative variables.

In the second phase of the analysis, bivariate inferential analyses were performed using contingency tables via Pearson’s Chi-squared association test or Fisher’s exact test, with a statistical significance level of *p* < 0.05, to determine the significant relation between the use of Grindr and the rest of the variables analyzed.

Furthermore, a multiple logistic regression model was constructed to determine the factors associated with the risk of STIs among Spanish Grindr users. The independent variables that were included in the logistic regression model were those that had previously demonstrated a significance value of *p* ≤ 0.2 in the univariate analysis. The final model included those that demonstrated a level of significance of *p* ≤ 0.05 in Wald’s test, in the automatic method “backward Wald”. The estimator obtained was the Adjusted Odds Ratio (AOR) with a corresponding 95% Confidence Interval (95% CI).

Questionnaires were checked for errors before the data were entered, coded, and analyzed, using the Statistical Package for the Social Sciences software (IBM Corp. Released 2023. IBM SPSS Statistics for Windows, version 24.0. Armonk, NY, USA: IBM Corp.) under the University of Zaragoza’s license.

### 2.5. Ethical Considerations

This study was reviewed and approved by the Aragón Research Ethics Committee, Spain (CEICA; project identification code PI18/327). This research also fulfilled the rules and ethical principles of the Declaration of Helsinki. Participation in this study was anonymous and voluntary, and all the participants consented via an online informed consent document before participating in this study. Moreover, data were anonymized.

## 3. Results

### 3.1. Sociodemographic and Behavioral Characteristics

The sample included 881 MSM aged between 16 and 75 years old (M = 33.9; SD = 9.98), with most of them between 30 and 50 years old (417; 47.3%). Of these participants, 72.8% were born in Spain, 72.5% reported completing higher education, 77.4% were employed, and 54.6% declared an income ≥ 1200 EUR/months. Most of them lived with their family/friends or in shared apartments (42.8%) and lived in cities with ≥1,000,000 habitants (Table 1).

Regarding behavioral characteristics, 85.9% described themselves as gay/homosexual, followed by bisexual (13.1%), and heterosexual (1%). The majority (49.1%) identified themselves as versatile (insertive and receptive roles) in sexual anal intercourse, and nearly half of them declared having had sex with casual sexual partners in the past 12 months (40.5%). A total of 10% in the sample affirmed that they sometimes/usually participated in Chemsex, 25.8% admitted that they consumed drugs while they had sexual intercourse, and 7.7% declared that they usually used PrEP. Finally, 49% reported having had STIs in the past (Table 2).

Regarding the use of Grindr by participants, 33.4% (n = 294) were moderate users, and 66.6% (n = 587) were intensive users. The association between Grindr use and sociodemographic and behavioral characteristics can be seen in Table 1 and Table 2. The sociodemographic variables that were associated with Grindr use were cohabitation situation and city size. Intensive Grindr use was observed among participants who lived alone (72.1%; *p* = 0.001) and among those who lived in cities with more 1,000,000 habitants (69%; *p* = 0.049). Concerning behavioral characteristics, participants with multiple casual sexual partners reported intensive app use (76.2%) in comparison with those who reported moderate use (23.8%; *p* < 0.001). In addition, a higher proportion of intensive app use was observed among those who participated in Chemsex (77.3%; *p* = 0.031) and among those who were previously diagnosed with sexually transmitted infections (69.9%; *p* = 0.046).

### 3.2. Sexually Transmitted Infections

The majority of STIs reported by the participants were gonorrhea (n = 184; 27.3%), syphilis (n = 149; 22.1%), and chlamydia (n = 91; 13.5%). However, regarding Grindr use, the frequency of declared STIs presented differences, and most of them were not statistically significant. Moderate Grindr users reported higher proportions of HIV (n = 31; 38.8%), human papilloma virus (HPV) (n = 27; 33.3%) and hepatitis (n = 19; 30.2%), while intensive Grindr users reported higher proportions of other STIs (crabs, genital herpes, and others) (n = 21; 77.8%), gonorrhea (n = 172; 73.9%), and syphilis (n = 107; 71.8%) (Table 3).

### 3.3. Attitudes toward HIV Testing

Regarding users’ attitudes toward HIV testing, 11.9% (n = 80) reported having tested positive for HIV (Table 3). A higher proportion of moderate Grindr users reported never having been tested for HIV (39.2%), whereas intensive users reported a higher frequency of HIV testing (69.7%; *p* = 0.045). The most popular reason for HIV testing for moderate users was “To confirm that they had the same HIV status as their partner in order to stop using condoms” (45.6%), and for the intensive users, it was “I was recommended to be tested” (74%). When we asked those who had never been tested for HIV as to the reason why, moderate users stated that “There are no places to get tested close to where I live” (50%) and “Test is too expensive for me” (50%); however, intensive users reported, “I am afraid to talk about my sexuality” (85.7%) (Table 4).

Analyzing the perceived risk of HIV infection, a low self-perceived risk of HIV infection was reported among moderate users (34.4%), whereas intensive users perceived themselves as having a moderate risk of HIV infection (71.2%; *p* = 0.048). For moderate users, the main reason for this low self-perceived risk was, “I am in a closed relationship” (65.6%). Conversely, for intensive users, the reason for self-perceiving a moderate risk of HIV infection was “I prefer not to know my HIV status (whether or not I have HIV)” (78.6%) (Table 4).

Multivariate logistic regression analysis, adjusted for age, living situation, and settlement size (variables with a significance value of *p* ≤ 0.2 in the univariate analysis), demonstrated different factors associated with the risk of STIs in the sample of Spanish Grindr users studied. MSM who were intensive Grindr users had an almost one-and-a-half-time higher risk of contracting STIs (AOR 1.36; 95% CI 1.15–1.91). The risk for those who had sex under the influence of drugs was almost two and a half times higher (AOR 2.48; 95% CI 1.73–3.55) and four times higher (AOR 4.1; 95% CI 2.04–8.22) for MSM who used PrEP. Those who had casual sex presented an almost two-time higher risk of contracting STIs (AOR 1.82; 95% CI 1.14–2.90), and the risk for those who alternated between regular and casual partners was slightly more than one and a half times higher (AOR 1.76; 95% CI 1.10–2.94) (Table 5).

## 4. Discussion

The prominent frequency of Grindr use among our sample of Spanish MSM speaks to, as other authors have highlighted, the growing popularity of this app worldwide, raising important questions that must be answered regarding MSM’s sexual health [4,9,27,28].

Compared to other studies in similar contexts, we found some differences. It is important to highlight the fact that our sample size was larger than those in other published studies (881 vs. 457) [29].

We found specific patterns of Grindr use in our population. The observed trends indicate that the use of the app was primarily intensive at all ages, being significantly intensive at younger ages [14]. Additionally, higher levels of education, unemployment situations, and lower income levels intensified Grindr use.

Intensive use was also related to living alone, which indicates changes in MSM’s socialization processes, as others have found [4]; this finding is interesting when considering assertions made by other authors about the existence of a particular means of interaction in these kinds of users, serving a particular form of community function, with opportunities to explore their own identities and fulfill other emotional and relationship-oriented needs.

Moreover, living in a town with fewer than 100,000 habitants was related to intensive Grindr use—a finding that requires closer attention in the context of unique specificities in other countries, for example, in those with rural communities. Studies not exclusively centered on Grindr use have shown the importance of reducing the vulnerability associated with these factors in the respective populations studied, such as when opportunities to access education and engage in healthy sexual interactions are scarce [30].

Additionally, our results show a self-reported risk for a higher number of STIs among intensive Grindr users. Specifically, we found differences in age, such that with the increase in age, the risk of having been diagnosed previously appears more frequent. Consistent with previous reports, Grindr users in our study were more likely to test positive for STIs [12,14,18,31].

Intensive Grindr users acknowledged having a moderate risk of HIV infection because they prefer not to know their HIV status. Moderate Grindr users perceived themselves to be at a low risk of HIV infection because they reported maintaining a closed relationship, and as such, were also less likely to undergo testing for HIV, although it should be noted that 36% of users reported having had sex with both regular and casual partners in the past year [11,12,13,14]. For this reason, individuals who believed they were at a moderate risk of HIV were more likely to adapt their behavior in order to reduce such a risk, in contrast to those who self-perceived themselves to be at a low risk being unlikely to adopt healthy or protective behaviors [32]. It is important to highlight that the self-perceived and actual risk of HIV are not directly associated with each other in the MSM population [32,33]. Moreover, there are individuals who may perceive themselves to be at no risk or at a low risk of HIV [34], and engaging in sexual practices that involve the risk of contracting HIV may not perceived as risky behavior [33], leading to the underestimation of self-perceived risk of HIV infection.

Importantly, almost half of the Grindr users (49%) declared having had at least one STI; of these, 11.9% reported having had HIV. Despite this, 14.2% of the users and participants in this study had never been tested for HIV—higher levels than in other published studies [35,36]. Nevertheless, these results were lower compared to those in a report from the European MSM Internet Survey (EMIS-2017) in the Spanish population, which stated that 19.4% of MSM had never been tested for HIV [37].

Our study complements previous studies conducted on the topic and provides the reason as to why HIV testing is not performed. In general, the reason participants reported having taken their most recent HIV test was “I get tested frequently”; however, moderate users underwent testing because “I was tested to confirm that had the same HIV status as my partner to stop using condoms”, and intensive users underwent testing because “I had a risky practice and wanted to have it done”. Conversely, those who had never been tested for HIV stated, “I have not had any risk practice so far”. However, for moderate users, the main reasons were related to the availability of testing, with reasons including “The are no places to get tested close to where I live” and “Test is too expensive for me”. For intensive users, the reason for not having been tested was motivated by fear of expressing their sexuality—“I am afraid to talk about my sexuality”. These reasons should be taken into account in public health and should be considered in the development of new prevention and self-care strategies.

In this study, the use of the most popular dating app, Grindr, was assessed in 881 Spanish MSM. On the one hand, we wanted to describe the usage of Grindr patterns according to sociodemographic and behavioral characteristics and identify the prevalence of STIs. On the other hand, we wanted to investigate attitudes toward HIV testing in a sample of Spanish MSM. From these results, it is evident that Grindr is just another tool that is increasingly known and used by sexual minority populations to contact and interact with potential sexual partners.

Our results show that Grindr users reported behaviors that placed them at a greater risk for STIs and HIV. However, differences were found according to the pattern of use. In our sample, intensive Grindr users were characterized by living alone and in large cities. As in another study [4], these findings indicate changes in socializing, especially among urban MSM, with technology imposing itself over the traditional forms of socializing, such as gay bars and gay neighborhoods. We also found that intensive users had more casual partners, participated in Chemsex, and reported more STIs. Although they were more likely than moderate users to report more STIs, they also had a better attitude toward HIV testing, indicating that Grindr could serve as a public health tool for STI control [38]. Public health services should be attentive to new emerging phenomena in Spain, such as Chemsex, substance use, and PrEP use, which affect populations vulnerable to HIV and other STIs, such as MSM [19,24,39]. All of these factors allow for the identification of app user profiles to which future interventions in prevention and health promotion should be directed.

Compared to other studies in similar contexts, our study contends with certain differences. For example, our sample size was larger than those in other published studies [29]. However, there are no previous studies assessing usage patterns and sociodemographic characteristics, such that we have analyzed, specifically in the Spanish context. Moreover, our results show greater vulnerability behind Grindr use, such as in cases among those who live alone and in large cities. Along these lines, previous studies have shown the importance of reducing vulnerability as a result of these factors in the respective populations studied [30]. Additionally, our sample included very few heterosexuals compared to other studies [16,29]; nevertheless, it is possible that their use of dating apps is increasing, and future research should better explore their usage patterns and not obviate them in research. 

Promoting regular HIV testing allows for the early identification and treatment of HIV among at-risk MSM, and identifying their HIV prevention needs allows for more effective interventions. The surge in dating apps and their associations with risky behavior offers opportunities for the development of unique prevention messages about health promotion and education [14,40]. In this regard, the opportunity should be taken to create health care points and health literacy on Grindr to improve the uptake of STI testing and to be able to detect and prevent STI outbreaks in the MSM population. Previous studies have already positively evaluated the use of Grindr advertisements for the recruitment of at-risk populations [4,9,38,41,42,43]. Furthermore, according to our results, the use of Grindr for user recruitment from those participating in new sexual health trends, such as Chemsex, substance use, or PrEP use, should be taken into account for implementation intervention programs, such as harm reduction campaigns [42,43,44].

There are several limitations to this study that need to be taken into account when interpreting the results. The first of these is related to the sample itself because the census population of Spanish MSM is unclear; thus, our findings might not be generalizable to the national population. According to participant recruitment, this study was available for 48 h; therefore, we may not have consistently captured both moderate and intensive Grindr users. Furthermore, we found under-represented categories as a result of online dissemination. Additionally, is important to consider that using an online questionnaire might have resulted in the under-reporting of sexual behavior to match social desirability. Second, some aspects related to the results reported concerning ever having had STIs should be discussed. This study had a high percentage of participants who reported STIs, specifically HIV prevalence; therefore, the attitude to HIV testing and other STIs might have been overstated. Finally, the data can also be skewed because there are other geospatial networking app platforms that we have not studied, and these should be considered.

Future studies should consider assessing on-demand PrEP use, Chemsex participation, and substance use, and they should incorporate objective measurements of the impact of dating app use on MSM health.

## 5. Conclusions

Grindr use appears to be intensive among MSM in Spain, and it is related to sociodemographic and behavioral characteristics. Unique traits such as living alone and living in towns and cities with fewer than 100,000 habitants or having casual sexual partners, engaging in Chemsex and being previously diagnosed with an STI define the sexual health trends in MSM Grindr users in Spain.

With some limitations, age, sexual partners, intensive Grindr users and PrEP use were associated with the risk of STIs in the sample of Spanish Grindr users studied. Nevertheless, intensive Grindr users also undergo testing more frequently, although efforts are needed to improve these results.

## Figures and Tables

**Table 1:** Sociodemographic characteristics according to Grindr users participants (n = 881).

Variables	n	%	Grindr Users		
			% Moderate	% Intensive	*p*
**Age (years)**					0.348
<30	297	33.7	31.3	68.7	
30–50	417	47.3	32.1	67.9	
50–75	61	6.9	39.3	60.7	
**Place of birth**					0.522
Spain	641	72.8	34	66	
Other	240	27.2	31.7	68.3	
**Education level**					0.444
College degree and above	639	72.5	32.2	67.8	
Vocational training	186	21.1	35.5	64.5	
Secondary school and below	56	6.4	39.3	60.7	
**Employment**					0.174
Retired	7	0.8	71.4	28.6	
Student	145	16.5	31	69	
Employed	682	77.4	33.6	66.4	
Unemployed	47	5.3	31.9	68.1	
**Monthly incomes**					0.524
≥1200€	481	54.6	31.8	68.2	
700–1200€	219	24.1	37	63	
≤700€	181	20.5	33.1	66.9	
**Living Situation**					0.001
Stable partner	196	22.2	43.9	56.1	
Others (Family, shared apartment)	377	42.8	32.4	67.6	
Alone	308	35	27.9	72.1	
**Settlement size (habitants)**					0.049
<10,000	32	3.6	43.8	56.3	
10,000–100,000	62	7	30.6	69.4	
100,000–500,000	101	11.5	42.6	57.4	
500,000–1,000,000	70	7.9	38.6	61.4	
>1,000,000	616	69.9	31	69	

*p*: *p*-value: Pearson’s Chi-square and Fisher’s exact test statistical significance *p* < 0.05.

**Table 2:** Behavioral characteristics according to Grindr users (n = 881).

Variables	n	%	Grindr Users		
			% Moderate	% Intensive	*p*
**Sexual Role**					0.921
Active/insertive	244	27.7	33.2	66.8	
Versatile (insertive and receptive)	433	49.1	33.9	66.1	
Passive/receptive	204	23.2	32.4	67.6	
**Sexual Orientation**					0.088
Heterosexual	9	1	33.3	66.7	
Bisexual	115	13.1	24.3	75.7	
Gay/homosexual	757	85.9	34.7	65.3	
**Sexual Partners (past 12 months)**					<0.001
Regular sexual partners	73	8.3	60.3	39.7	
Casual sexual partners	357	40.5	23.8	76.2	
Both regular/casual	316	35.9	35.8	64.2	
**Chemsex participation**					0.031
Never	793	90	34.6	65.4	
Sometimes/usually	88	10	22.7	77.3	
**Sex while under the influence of drugs**					0.807
No	654	74.2	33.6	66.4	
Yes	227	25.8	32.6	67.4	
**PrEP use**					0.422
Never	813	92.3	33	67	
Usually	68	7.7	38.2	61.8	
**STIs**					0.046
No	449	51	36.5	63.5	
Yes	432	49	30.1	69.9	

STIs: Sexual Transmitted Infections; Chemsex: Intentional sexual practices under the effect of psychoactive drugs, in a group for a prolonged duration; PrEP: Human immunodeficiency virus pre-exposure prophylaxis; *p*: *p*-value: Pearson’s Chi-square and Fisher’s exact test statistical significance *p* < 0.05.

**Table 3:** Sexually Transmitted Infections reported by the participants (n = 432; 49%).

STIs	n	%	Grindr Users				
			Moderate		Intensive		
			n	%	n	%	*p*
HIV	80	11.9	31	38.8	49	61.3	0.285
HPV	81	12	27	33.3	54	66.7	0.994
Chlamydia	91	13.5	27	29.7	64	70.3	0.566
Syphilis	149	22.1	42	28.2	107	71.8	0.141
Gonorrhea	184	27.3	48	26.1	172	73.9	0.018
Hepatitis (A, B, C)	63	9.3	19	30.2	44	69.8	0.575
Others: Crabs, genital herpes, etc.	27	4	6	22.2	21	77.8	0.212

STIs: Sexual Transmitted Infections; HIV: Human Immunodeficiency virus; HPV: Human Papillomavirus. *p*: *p*-value: Pearson’s Chi-square and Fisher’s exact test statistical significance *p* < 0.05.

**Table 4:** Attitudes toward HIV testing of the Spanish Grindr users (n = 881).

Variables	n	%	Grindr Users		
			% Moderate	% Intensive	*p*
**Have you been tested for HIV?**					**0.045**
Yes, in the last 12 months	545	61.9	30.3	69.7	
Yes, but not in the last 12 months	211	24	37.9	62.1	
Never	125	14.2	39.2	60.8	
**The last time you were tested, was because:**					
I had a risky practice and wanted to have it done	225	26.1	29.8	70.2	
I had symptoms that could suggest HIV infection or AIDS	47	5.4	34	66	
I was forced to be tested	18	2.1	38.9	61.1	
I was recommended to be tested	96	11.1	26	74	
I was tested to confirm that I had the same HIV status as my partner to stop using condoms	57	6.6	45.6	54.4	
I get tested frequently	410	47.5	30.2	69.8	
Other	10	1.1	30	70	
**“I’ve never been tested for HIV” because:**					
I have not had any risky practice so far	59	25.5	42.4	57.6	
I have risky practices, but I don’t think I have been infected	35	12.2	34.3	65.7	
I don’t think I am HIV positive because my partner is HIV negative	3	1.3	33.3	66.7	
I’m afraid of them knowing that I have sex with men	10	4.3	40	60	
I am afraid that they will think I am HIV positive	8	3.5	25	75	
I am afraid to talk about my sexuality	7	3	14.3	85.7	
I do not want to be judged for my sexuality and sexual practices	9	3.9	33.3	66.7	
I am afraid of getting a positive test result	33	14.3	33.3	66.7	
I could not bear the stress of waiting for the test result	10	4.3	40	60	
I am afraid that there is not enough confidentiality	15	6.5	40	60	
There are no places to get tested close to where I live	12	6.5	50	50	
Test is too expensive for me	8	3.5	50	50	
It takes too long to get the test results	4	1.7	25	75	
I would prefer not to know my HIV status (whether or not I am HIV positive)	7	3	28.6	71.4	
I can be tested in a while without losing treatment options	11	4.8	45.5	54.5	
**Self-perceived risk of HIV infection**					**0.048**
At low risk of HIV infection	718	81.5	34.4	65.6	
At moderate risk of HIV infection	163	18.5	28.8	71.2	
**“You are at low risk of HIV infection”, please tell us why:**					
I have not had unprotected sex.	396	45.5	30.8		
I am in a closed relationship	64	7.3	65.6		
My sexual partner is HIV negative	79	9.1	46.8		
My sexual partner is not at risk of infection	45	5.2	53.3		
I have never had more than 2 or 3 relationships in which I did not use a condom regularly	242	27.8	30.6		
I have started taking PrEP (Pre-Exposure Prophylaxis)	46	5.3	28.3		
**“You are at moderate risk of HIV infection”, please tell us why:**					
I have been unable to make an appointment for HIV testing	16	8.4		68.8	
It takes too long to know the results of the test	25	13.1		68	
I am not sure where I need to go for testing	29	15.2		69	
I am afraid of a positive test result	69	36.1		66.7	
I prefer not to know my HIV status (whether or not I have HIV)	14	7.3		78.6	
I feel sad or depressed	38	19.9		73.7	

*p*: *p*-value: Pearson’s Chi-square and Fisher’s exact test statistical significance *p* < 0.05.

**Table 5:** Factors associated with risk of Sexually Transmitted Infections among Spanish Grindr users (n = 881).

	AOR	(95% C.I.)	*p*
**Age**			
	1.05	(1.02–1.06)	<0.001
**Sexual Partners (past 12 months)**			
Regular sexual partners	1		
Casual sexual partners	1.821	(1.14–2.9)	0.011
Both regular/casual	1.765	(1.10–2.94)	0.019
**Grindr User**			
Moderate	1		
Intensive	1.357	(1.15–1.91)	0.048
**Sex while under the influence of drugs**			
No	1		
Yes	2.483	(1.73–3.55)	<0.001
**PrEP use**			
Never	1		
Usually	4.1	(2.04–8.22)	<0.001

AOR: Adjusted Odd Ratio by age, living situation and settlement size; (95% C.I.): 95% Confidence Interval; *p*: *p*-value: Wald’s test p statistical significance *p* < 0.05.

## Data Availability

The datasets generated and analyzed during the current study are not publicly available due the fact that they constitute an excerpt of research in progress, but they are available from the corresponding author upon reasonable request.
